# “I Worry About My Kids’ Safety When They Visit”: Mothers’ Perceptions of Father/Child Post-Separation Contact in the Context of IPV

**DOI:** 10.1177/10778012231225232

**Published:** 2024-01-09

**Authors:** Leslie M. Tutty, Kendra L. Nixon, H. Lorraine Radtke

**Affiliations:** 1 Faculty of Social Work, University of Calgary, Calgary, AB, Canada; 2 Faculty of Social Work, University of Manitoba, Winnipeg, MB, Canada; 3 Department of Psychology, University of Calgary, Calgary, AB, Canada

**Keywords:** child access and contact, child custody, child visitation, intimate partner violence, violence against women

## Abstract

After separation because of intimate partner violence, fathers’ contact with children can be problematic. This mixed methods secondary analysis focused on 280 Canadian separated/divorced mothers who were 48.4% White, 45.1% Indigenous, and 6.5% Visible Minority. Of 176 fathers, 105 (59.7%) had regular visits and, 71 (40.3%) visited sporadically; 104 had no contact. Comments from half the mothers (54.3%) with regular father–child visits indicated worry, and 41.9% of all mothers perceived their children as sad/upset and another 14.5% as angry/acting out in response to visitation. Recommendations to address mothers’ and children's issues with respect to problematic father–child contact are provided.

Violence against women (VAW) is a significant global problem that results in injury, emotional harm, and, at worst, death (Dawson et al., 2009; World Health Organization, 2021). The abuse that male partners perpetrate on women takes many forms, most-often extending throughout the relationship. It includes physical, sexual, and emotional abuse, all usefully perceived as “coercive control” (Stark, 2007). Intimate partner violence (IPV) commonly continues after women leave abusive partners (DeKeseredy et al., 2004; Hardesty & Ganong, 2006) either through continued physical assaults (Brownridge et al., 2008; Rezey, 2020), sexual assaults (DeKeseredy et al., 2004) or controlling tactics (Hayes, 2012; Tutty et al., 2023).

Nevertheless, despite the potential for continued partner abuse during child exchanges, in a study of 70 U.K. family court cases with evidence of IPV, in most, father contact with children was perceived as both desirable and inevitable (Macdonald, 2016). More research about the nature of father/child contact in the context of IPV and its impact on mothers and children is needed and constitutes the focus of the current investigation.

## The Impact of Child Contact and Custody Exchanges

Even when protective orders are granted to the mothers because of serious nature of their partner's IPV, fathers are often given child visitation rights (Fleury-Steiner et al., 2016; Rosen & O'Sullivan, 2005). The necessary custody exchanges potentially provide opportunities to continue to air grievances, which may erupt into further violence (Holt, 2015; Morrison, 2015; Thiara & Humphreys, 2017; Varcoe & Irwin, 2004). For many mothers, father visitation is the only reason that they have any contact with abusive ex-partners (Hayes, 2017).

Some authors question the benefits of fathers having any child contact when IPV is a consideration (Elizabeth et al., 2012; Tolmie et al., 2009). Holt (2011, 2017) surveyed 219 mothers and held focus groups/individual interviews with youth, mothers, and fathers, concluding that, not only were children not involved in decisions about father contact, but that child maltreatment including emotional, physical, and sexual abuse, often continued in contact visits with fathers. This conclusion is similar to Katz et al. (2020) who found that children may experience acute fear, physical health problems, and distress during visits, with some being emotionally, physically, and/or sexually abused. Detailing some of the dynamics that cause problems for children, Morrison (2015) interviewed 18 children (aged 8–14) and 16 mothers about father visitation in Scotland. Given the continued parental conflict, the children had to navigate emotionally fraught dynamics, such as being given messages to send to the other parent or having to avoid mentioning the other parent for fear of arousing anger. Besides the possibility of further abuse toward mothers, other problems around contact visits include fathers arriving late or not coming, being rigid about arrangements, or not addressing children's developmental needs (Holt, 2015, 2017; Reece, 2006). Taken together, these studies identify a need to carefully review what constitutes the best interests of the children.

Some studies have focused on the consequences of father visitation for children. Hunter and Graham-Bermann (2013) examined the relationship between child adjustment and father contact in 219 children whose fathers had used IPV against their mothers. Thirty percent had had no contact in the previous year (similar to Forssell & Cater, 2015), and child problems were related to the frequency of IPV but not to contact per se. The authors speculated that child contact with a non-violent or less violent father or father figure could buffer the effects of children's behavior problems. Consistent with this supposition, in a study with 66 mothers and their children who had stayed in a VAW shelter, Jouriles et al. (2018), concluded that more contact with fathers was correlated with further IPV and father–child aggression, the latter negatively affecting children's conduct problems. In a recent Canadian study, most of the 59 child participants commented on the control and violence that their fathers had exerted on the families pre-separation; however, their post-separation contact was limited or non-existent, which they perceived as positive (Lapierre et al., 2022). Thus, it cannot be taken for granted that children benefit from or even desire contact with their fathers post-separation.

A worrisome finding is that professionals may overlook problems associated with child contact. Thiara and Humphreys (2017) interviewed 45 mothers and 52 children, concluding that disagreements about child contact arrangements provided a vehicle for fathers to continue to harass the women and undermine her mothering, but that this ongoing abuse was rarely seen as important by the professionals in the women's lives. In a recent study in Manitoba, Canada, 20 parents, three youth, and 24 criminal court and community agency staff were asked about the intersection of criminal and family courts in IPV cases and the impact on children (Hoffart et al., 2022). Neither family nor criminal courts were seen as consistently considering IPV as critical in rulings that affect women and children. Similar findings were reported by Coy et al. (2015) who conducted interviews with 34 mothers in the United Kingdom. Most of the women (69%) perceived judges as dismissing their concerns about father–child contact, with no conditions attached in three quarters of these cases. While women viewed supervised contact as the safest alternative, this was rarely granted. Clearly, the majority of children of women leaving an abusive relationship will encounter problems associated with contact with their fathers but such problems will be ignored by relevant professionals.

Despite the experiences documented through research, most mothers in several studies wished their children to have at least some contact with their fathers (e.g., Coy et al., 2015; Humphreys & Harrison, 2003; Morrison, 2015). That children require a relationship with their fathers for normal development to occur is an issue that perhaps requires further exploration.

Nevertheless, given concerns about father–child contact after IPV, with access to a large number of abused mothers separated from partners, the current study explores women's perceptions about the effects of their partner's visitation on themselves and their children.

## Rationale for the Current Study

In summary, studies of child visitation and access are not common. Most are qualitative, with samples of from 12 to 39 (Coy et al., 2015; Elizabeth, 2017; Morrison, 2015; Tolmie et al., 2009), although some have larger samples (Hoffart et al., 2022; Holt, 2015; Hunter & Graham-Bermann, 2013; Lapierre et al., 2022; Thiara & Humphreys, 2017). Several quantitative studies have been published (Holt, 2011; Jouriles et al., 2018) but, regardless of methodology, few studies examine the circumstances when fathers have no contact with children (exceptions are Jouriles et al., 2018; Hunter & Graham-Bermann, 2013; Lapierre et al., 2022) and none differentiate when fathers are in regular versus sporadic contact.

Clearly, more research examining the experiences of a larger number of women and their children with diverse backgrounds are recommended and should include circumstances in which there is no contact between children and fathers. More comprehensive research that documents that serious nature of the IPV and the impact of the father/child visits on both mothers and children would be beneficial. To fill that gap, the current study utilizes data from the “Healing Journey Project,” a longitudinal study of women with a history of IPV across the Canadian prairie provinces. A key question for the study is, “When IPV was a factor in a divorce/separation, how do mothers experience and perceive their children responding to contact with fathers?” Our analysis explores child visitation arrangements of 280 women, whether regular, sporadic, or no contact, and their perceptions of those arrangements, including the impact on themselves and their children. Importantly, almost half of the women were of Indigenous origins, a group about which we know little with respect to child custody after IPV.

## Method

This mixed methods secondary analysis study included both quantitative and qualitative components (Sandelowski, 2000). The “Healing Journey Project” employed a convenience sample of 665 women who had experienced IPV and lived in the three Canadian provinces of Alberta, Saskatchewan, and Manitoba. The original study assessed characteristics of women impacted by IPV including mental health and general well-being (Tutty et al., 2021a), following them over 2.5 years (Tutty et al., 2021b), as well as examining their experiences of mothering (Ateah et al., 2019; Nixon et al., 2017). Data were collected in seven waves between 2005 and 2009. The criteria for inclusion were: minimum of 18 years of age; the most recent incident of IPV no sooner than 3 months and no longer than 5 years prior; commitment to stay in the study for the full 2.5 years; and no debilitating mental health issues. Honoraria ($50 CAN) were given to participants at each wave. The research protocols were approved by the Ethical Review Boards of the six associated universities (Universities of Manitoba, Calgary, Regina, Brandon, Winnipeg, and Lethbridge).

The IPV was measured by The Composite Abuse Scale (CAS) (Hegarty et al., 2005), a 30-item self-report measure with four subscales: Severe Combined Abuse, Emotional Abuse, Physical Abuse, and Harassment as well as a Total CAS score. The items ask whether partners took certain actions (past 12 months) and the frequency of such actions rated on a 6-point Likert scale of *never* (0), *only once* (1), *several times* (2), *once per month* (3), *once per week* (4), or *daily* (5), for a total possible score of 150 (Hegarty et al., 2005). The clinical cutoff for the entire scale is 3–7 (Hegarty et al., 2005). The CAS has strong criterion and construct validity, as well as internal reliability (α = .85); the subscales also have a Cronbach's alpha of .85 or above (Hegarty et al., 2005). Cronbach's alpha in the original Healing Journey Project is .93.

### Quantitative Data Analysis

The quantitative analyses described the demographic characteristics and extent of the IPV. Pearson's chi-square analyses were calculated on demographic variables based on whether fathers visited their children or not, with effect sizes calculated with Phi or Cramer's *V*. Standardized residuals identified category differences responsible for the statistically significant chi-square analysis (Field, 2009). Effect sizes were interpreted using Rea and Parker's (2002) suggested benchmarks of under .10 as a “negligible” association, between .10 and under .20 as a “weak” association, between .20 and under .40 as a “moderate” association, and between .40 and under .60 as a relatively “strong” association (p. 203). Continuous variables were compared with independent *t*-tests, and effect sizes were calculated as *r*-values (Field, 2009). According to Cohen (1988), *r*s of .2, .5, and .8 are the small, medium, and large reference values, respectively.

### Qualitative Research Analysis

Qualitative secondary analysis re-uses preexisting qualitative data from previous research (Heaton, 2008), an important option since qualitative studies often produce a wealth of data not used in the primary analysis. Secondary research is especially appropriate with vulnerable populations to prevent repeated questioning that might be traumatic (Long-Sutehall et al., 2010; Sullivan-Bolyai et al., 2005). Descriptive qualitative analysis is particularly appropriate for mixed methods research (Neergaard et al., 2009) and uses the practicality of the research question as the guiding principle, rather than epistemological confines of qualitative traditions such as grounded theory or phenomenology (Neergaard et al., 2009).

The current study used descriptive qualitative research (Neergaard et al., 2009; Sandelowski, 2000) to examine the visitation experiences of 280 mothers who had been abused by intimate partners as explained in responses to four open-ended survey questions: (1) whether the father visits the children; (2) if mother worries about their ex/partners or about their children's safety, (3) the effects of father–child contact on themselves, and (4) the effects of the father–child contact on their children. The interviewers read the questions and wrote down the women's responses. The first question was used to differentiate the types of child contact: Comments in response to the remaining questions constituted the major focus of the qualitative analysis.

The descriptive analysis followed established inductive content analysis processes (the meaning emerges from the quotes) (Elo & Kyngäs, 2008; Sandelowski, 2000). We identified the major themes (Graneheim & Lundman, 2004; Neergaard et al., 2009), categorized by the different types of visitations since these each have distinctive characteristics. As noted by (Nassaji, 2015), data collected qualitatively can also be analyzed quantitatively. This happens when the researcher first examines the qualitative data thoroughly to find the relevant themes and ideas and then converts them into numerical data for further comparison and evaluation (p. 130). Given the large number of comments available for analysis, it was deemed appropriate to examine the themes by calculating the proportions of themes regarding each survey question.

## Results

### Demographics

The current secondary analysis focused on 280 women (42.1% of the original 665) who were separated from their partners and documented whether the fathers of their children had child contact (*N* = 176, 62.9%) or not (*N* = 104, 37.1%) (see Table 1). According to the women, of the 176 fathers with contact, most (105, 59.7%) had regular visits, while 71 (40.3%) visited sporadically. Of the 104 fathers with no contact, the largest group (57, 54.8%) was simply documented as “no visits”; 47 (45.2%) fathers were not available for child contact because they were in jail (*n* = 12), lived far away (*n* = 13) or had restraining or no contact orders (*n* = 22).

**Table 1.** Demographics of Mothers and Father–Child Contact (*N* = 280).

Variable	Categories	No contact (*N* = 104)	Contact (*N* = 176)	Statistical test
Age in years (*N* = 278)		32.7 (*SD* = 7.9)	35 (*SD* = 7.5)	*t* = 2.4; *p* = .02*; Cohen's *d* = .30
Ex-partner age (*N* = 280)		35.4 (*SD* = 9.1)	37.4 (*SD* = 8.5)	*t* = 1.8; *p* = .07 *ns*
Total yearly income (past year) (*N* = 258)		$21,413 (*SD* = 27,024)	$24,426 (*SD* = 26,015)	*t* = 0.8; *p* = .38 *ns*
Woman's ethnic identity (*N* = 277)	Indigenous	51 (40.8%)	74 (59.2%)	χ^2^ = 3.5*; p* = .18 *ns*
	White	43 (32.1%)	91 (67.9%)	
	Visible minority	9 (50%)	9 (50%)	
Ex-partner ethnic identity (*N* = 275)	Indigenous	51 (31.1%)	90 (68.7%)	χ^2^ = 3.7*; p *= .16 *ns*
	White	41 (31.4%)	90 (68.7%)	
	Visible minority	10 (45.5%)	12 (54.5%)	
Age of oldest child Custody type (*N* = 280)
	No formal or unclear	38 (40.4%)	56 (59.6%)	χ^2^ = 6.4; *p* = .09 *ns*
	Preschooler	34 (47.9%)	37 (52.1%)	
	6–12 years	36 (36.7%)	62 (63.3%)	
	Teens (13–18 years)	22 (34.4%)	42 (65.6%)	
	Oldest adult but others below 18	12 (25.5%)	35 (74.5%)	
	Mother has sole custody	56 (42.7%)	75 (57.3%)	
	Joint custody	2 (6.5%)	29 (93.5%)	
	Custody in process	8 (33.3%)	16 (66.7%)	
Father visits children (*N *= 280)	Yes	0 (0%)	105 (100%)	
	Yes, but sporadically	0 (0%)	71 (100%)	
	No	57 (100%)	0 (0%)	
	Not available (in jail)	12 (100%)	0 (0%)	
	Not available (lives far away)	13 (100%)	0 (0%)	
	No contact or restraining order	22 (100%)	0 (0%)	
Combined abuse scores (*N* = 278)	CAS severe combined abuse	8.0 (*SD* = 7.5)	6.7 (*SD* = 6.4)	*t *= 1.5; *p* = .13 *ns*
	CAS emotional abuse	30.1 (*SD* = 14.6)	29.2 (*SD* = 13.5)	*t *= 0.5; *p* = .62 *ns*
	CAS physical abuse	14.0 (*SD* = 8.2)	10.8 (*SD* = 8.0)	*t *= 3.3; *p *= .001***; Cohen's *d* = .40
	CAS harassment	8.9 (*SD* = 5.4)	7.7 (*SD* = 5.1)	*t* = 1.9; *p* = .06 *ns*
	CAS total	59.6 (*SD* = 28.7)	53.9 (*SD* = 27.0)	*t *= 1.6; *p* = .11 *ns*

*Note.* CAS = Composite Abuse Scale; *ns* = not statistically significant.

*p* < .05 = *; *p* < .000 = ***

The women's average age was 34.2 years (*SD* = 7.7) while their ex-partners were an average of 36.7 years (*SD* = 8.8). Older mothers more often reported father/child contact (*t* = 2.4; *p* = .02*). The racial/ethnic identities of the women were 48.4% White, 45.1% Indigenous, and 6.5% Visible Minority. The ex-partners’ racial/ethnic identities were White (47.6%), Indigenous (44.4%), and Visible Minority (8.0%). Their average total income in the past year was $23,281 CAN; about half of the women had incomes below the poverty line for that time in the three Canadian provinces (DeRiviere, 2014). The oldest child ranged in age from being a preschooler to an adult but, in the latter case, the family had other children below the age of 18. Notably, there were no differences between the children's age and whether the fathers visited.

Scores on the CAS subscales and CAS Total were well above the suggested clinical cutoff scores for partner abusive behaviors. The women whose partners had no contact with their children reported the fathers using significantly more serious Physical Abuse (*t* = 3.3; *p* = .001***), a relatively strong effect, compared to the women whose partners had contact.

### Mother's Comments Regarding Child Visitation

The qualitative analysis of the mother's comments occurred independently for each type of father–child contact. Within each of these, the analysis first addresses the mothers’ comments pertaining to child visitation in the context of the IPV, followed by how the contact/lack of contact affected them (the mothers) and, finally, their perceptions of how contact/no contacted affected their children. Some mothers described supervised father/child visits and whether these worked as anticipated. As a possible important strategy to improve the quality of child contact for all parties, it was important to document this information.

*No father contact.* As mentioned, 104 fathers had no child contact. As is apparent in Table 2, women whose partners lived far away (*n* = 10) were the least likely to worry. Nevertheless, one mother worried about her ex-partner abducting the children, noting: “I’m very worried about the kids being abducted. On the reserve, they can be moved from house to house and the law cannot go on the reserve to find them.” Of the three mothers who expressed concerns for their own safety, one clarified: “I know he is looking for me. He will hurt me if he finds me.”

**Table 2.** Mother's Worries About Ex-Partner, Self, and Children in Context of IPV by Form of Father–Child Contact.

	Not worried	Worried about self	Worried about kids	Child abduction	Number of comments ^a^
No contact (*n* = 57)	24 (38%)	28 (44%)	4 (6%)	7 (11%)	63
No contact: father lives far away (*n* = 13)	10 (71.4%)	3 (21.4%)	0 (0%)	1 (7%)	14
No contact: father in jail (*n* = 12)	7 (50%)	7 (50%)	0 (0%)	0 (0%)	14
No contact: restraining order (*n* = 22)	7 (29.1%)	11 (45.8%)	1 (4.2%)	5 (20.8%)	24
Sporadic contact (*n* = 71)	38 (51.3%)	28 (37.8%)	7 (9.5%)	1 (1.4%)	74
Regular contact (*n* = 104)	38 (35.2%)	50 (46.3%)	16 (14.8%)	4 (3.7%)	108

a Some mothers mentioned more than one issue; some mothers did not comment. IPV = intimate partner violence.

Even with little or no contact, some mothers significantly worried about themselves or their children. Of the 12 mothers with partners in jail, while seven denied being currently concerned about their partners, eight worried about their safety in future when their ex-partner is released from prison, stating, for example, “When he gets out of prison, he will be gunning for us” and “When he gets out, he told me that he is coming.”

Of mothers with restraining or no contact orders (*n* = 22), seven women were not worried, but 11 were concerned for their safety commenting, for example, “I haven’t seen him but it's the fear of the unknown when/if I do” and “Really worried because of the threat to kill me. Always wondering what he will do next.” Three were concerned that restraining orders are not effective, for example, “I don’t know where he is. He is not concerned about being arrested or going to jail.” Three mentioned that ex-partners had breached these orders in the past. Five mothers were concerned about their ex-partners abducting their children: “I’m very worried about him taking the kids. He would use them to manipulate me back into the relationship or take children away as punishment” and “Scared he’ll come to get my child when he's outside playing.”

Of the 57 mothers with no specified reason for fathers not having contact with their children, 63 comments of a little more than one-third of them (38%) indicated that they were not worried. However, 28 comments (44%) related to women's fears of their own safety, such as: “My partner's mental instability is unpredictable. He is stalking me” and “He got away with five assault charges and told me, if he catches me, he’ll kill me and the kids. I know he will.” A final seven comments (11%) referred to the possibility of the fathers abducting the children, such as: “I worry he will abduct my son” and “He kidnapped the girl once right out of our yard.”

In summary, when fathers had no child contact, mothers whose ex-partners lived far away were the least worried. Women whose partners were in jail, women who did not specify a reason for the fathers not visiting the children, and women with restraining orders were similarly worried about their own safety including a total of 24 comments about their partners seeking their whereabouts or stalking them and six with explicit fears about their partner's intentions to kill them: “I worry about it frequently. When he attacked and tried to kill me, my six-year-old was there.” Two women commented about child abuse or her partner's improper behavior toward their daughter: “He was very inappropriate when he did see her. He said lewd things about me to her” and “I knew there was child abuse, but he still had custody rights.”

*The impact of fathers with no contact on mothers.* Regarding how the mothers were affected by their ex-partners having no contact with their children (see Table 3), the most-often reported reaction (103 comments) was feeling safe or happy (43, 41.3%), for example, “I feel very relieved as he hurt my son very badly at one time” and “I am relieved that he's not in the picture and hasn’t initiated seeing my daughter: Hasn’t seen her since she was four years old.” Another 22 (30.8%) comments found the lack of contact difficult or worrisome, for example, “I feel bad my son doesn’t have a father—especially because he's a boy” and “Difficult with my children's situations in trying to balance making safe decision for them while wanting them to have contact if and when the time is appropriate.” A small proportion (14, 13.5%) commented about the stresses of being a single parent, for example, “I bear all the responsibility—financial and otherwise. I have lots of financial fears” and “I am the mother, father, sole provider financially. I have a lot of stress.” Another 14 (13.5%) found that the lack of contact had no impact on them. Thus, most mothers reported a positive or neutral impact from the lack of contact, while some had concerns related to possible negative effects of father absence or the responsibilities of single parenthood.

**Table 3.** Impact on Mother of Father–Child Contact.

	No impact	Safe/happy	Difficult/worried	Stresses of single parenting	Irregular visits	Number of comments^a^
No contact (*n* = 105)	14 (13.5%)	43 (41.3%)	32 (30.8%)	14 (13.5%)	1 (0.9%)	104
Sporadic contact (*n* = 71)	13 (17.1%)	21 (27.6%)	20 (26.3%)	15 (19.7%)	7 (9.2%)	76
Regular contact (*n* = 104)	7 (6.7%)	21 (20%)	57 (54.3%)	9 (8.6%)	11 (10.5%)	105

a Some mothers mentioned more than one issue; some mothers did not comment.

*The impact of fathers with no contact on children.* Of the 99 comments about the impact of no father contact on the children (see Table 4), the largest proportion of mothers (28, 28.3%) perceived their children to be happy or relieved, for example, “Now that he is gone, they seem happy. They don’t seem to want a connection with him” and “Child will eat, sleep, and play now. When I was with my ex, he would do none of that. Still afraid to sleep on his own. Having me as sole parent is better for my son.” Another 23 (23.2%) of the mothers’ comments described the children as missing their fathers, for example, “They wish he would grow up and be their father” and “The boys want to see their dad. The oldest is heartbroken. He asks what's ‘wrong with me?’”

**Table 4.** Mother's Perceptions of Impact on Children of Father Contact.

	No impact	Happy	Sad/upset	Misses father	Angry	Upset re: irregularity	Want no contact	Number of comments^a^
No contact (*n* = 105)	19 (19.2%)	28 (28.3%)	17 (17.2%)	23 (23.2%)	5 (5.1%)	0 (0%)	7 (7.1%)	99
Sporadic contact (*n* = 71)	12 (14.1%)	14 (16.5%)	23 (27.1%)	14 (16.5%)	7 (8.2%)	10 (11.7%)	5 (5.9%)	85
Regular contact (*n* = 104)	10 (8.5%)	28 (23.9%)	49 (41.9%)	6 (5.1%)	17 (14.5%)	4 (3.4%)	3 (2.6%)	117

a Some mothers mentioned more than one issue or have several children; some mothers did not comment.

Smaller proportions of mothers (19, 19.2%) perceived their children as unaffected by not seeing their fathers, six because the children were too young. Other mothers (17, 17.2%) saw their children as sad or upset, for example, “They have mixed feelings about their father. One minute they hate him and the next they love him” and “Not knowing why she doesn’t see him. Thinking he doesn’t want to see her. Makes her sad.” Others saw their children as either wanting no contact with fathers (7, 7.1%) or angry/acting out (5, 5.1%). Of the former group: “His oldest son does not want anything to do with him.” Of the latter group: “In the beginning my oldest son experienced anger and temper tantrums. He is more accepting now but still has a lot of anger towards his father” and “Oldest child (age 9) threatens to kill the family and himself (they have been through counseling for his suicide ideation). Bites, kicks, screams, and swears at me and my middle child.” Two mothers mentioned their children receiving counseling, the one in the last quote and the other for signs of having been abused by her father. In short, most mothers reported that their children had responded positively to the lack of contact, with some desiring it, but a sizable minority reportedly missed their father or were upset.

### Father–Child Contact Sporadic

Seventy-one mothers described their children's fathers as visiting only sporadically. Of these, five had restraining orders against the ex-partners, four men were in prison (although one mother took the children to visit), and three lived far away. A little more than half of the comments (38, 51.3%) indicated no worries about themselves or their children. However, slightly more than one-third of the comments (28, 37.8%) indicated worry about their own safety, for example: “I’m afraid he’ll approach my son to get to me. He's been in jail before and I’m afraid he’ll take revenge on me for putting him there” and “He told me that he’d find a way to locate and kill me, so that's a constant worry.” Seven (9.5%) expressed concerns for their children, for example, “I worry about my kids’ safety when they visit. Is he going to be drinking? Is he going to be taking care of them?” and “I worry he may be abusive with children.” Only one comment referenced a fear that the ex-partner would abduct their children: “I’m always worrying about the children's safety; if ex is watching them and is he going to return them.” Thus, a sizable minority of mothers found even sporadic visits to be worrisome.

*The impact of father's sporadic contact on mothers.* Over one-quarter of the comments (21, 27.6%) indicated that the mothers were happy or felt safe with sporadic contact, for example, “It was good. He was out of my life, and I got to raise the girls on my own” and “I’m fine with it. I don’t fear he will try to take my son. He knows I’m a good mom.” In contrast, another 20 (26.3%) indicated worry/stress, for example, “It's very hard. He's rigid and opinionated. Does numerous things to irritate me like dressing the children without bathing them” and “When he's with them I worry what he’ll say and if he's drinking.” A further 15 (19.7%) were about the difficulties of being a single parent, 13 (17.1%) noted no impact and 7 (9.5%) mentioned difficulties with irregular visits. In short, a sizable proportion of mothers reported positive or no impact on them, while many found the sporadic visits difficult and of concern.

*The impact of father's sporadic contact on mothers.* Regarding the mother's perceptions of the impact on the children, a little over one-quarter of the 85 comments (23, 27.1%) indicated that their children seemed sad or upset about the visits, for example, “My oldest daughter prefers not to visit with her father as she remembers him abusing me and he kidnapped her and kicked both of us. She remembers being returned to me by the police”; “Disappointing. Unstable and insecure. Scared to be left alone”; and “Kids are hurt—feel dad doesn’t want them. They feel caught in the middle.” Another 14 (16.5%) reported that their children missed their fathers, for example, “The two middle children would like to have a father/son relationship, but their dad makes this difficult, and sometimes the boys have to hang-up on him” and “Youngest misses her dad; loving child, with a temper. She cries for him still, three years later.” A comparable proportion of mothers (14, 16.5%) reported that their children were satisfied with the visits, for example, “My 12-year-old has a good relationship with his father” and “Youngest seems to be okay and accepting.”

Twelve mothers (14.2%) reported no impact on their children, although four of them were too young to understand. Another 10 (11.9%) of the mother's comments reported negative effects associated with the irregularity of the visits, for example, “The younger one, it's written all over her face, she calls him a lot and he won’t call back for a couple of weeks” and “My oldest and her dad are fine together. He doesn’t come over much and my daughter asks, ‘Where's my dad?’” A small proportion of the comments (5, 5.9%) were that the children wanted no contact with their fathers: “They are free to call him whenever they want but they don’t” and “On occasion he’ll see his father but wishes he didn’t have to.” Finally, seven comments from mothers (8.2%) described their children as angry or acting out, externalizing symptoms that are of significant concern, for example, “They seem very angry and upset. It is affecting their behavior at home and at school”; “I notice big changes in my youngest son's personality after he visits his father. He becomes whiney, stubborn, aggressive, and loud”; and “Very rebellious. Confrontational because they don’t have control.” None of the mothers mentioned that any of these children were receiving counseling. To summarize, while about one-third of the mothers reported that their children were satisfied with or not impacted by sporadic visits with their fathers, the majority of mothers reported negative consequences.

### Fathers in Regular Contact With Children

Of the 104 fathers described as being in regular contact with their children, 59 (56.7%) had contact about once a month and 46 (44.2%) had contact weekly or biweekly. Of these mothers, seven reported having restraining orders (one recently expired) that restricted their ex-partners from contact with them but allowed child contact. Of 108 comments, a little more than one-third of the mothers (38, 35.2%) were not worried about the father/child visits. However, in contrast, another almost half (50, 46.3%) worried about their own safety, with comments ranging from unspecified or vague concerns such as: “He retaliated. He has violated my privacy many times”; to more specific threats: “He said he’d always find me, and the fact that he has access to the kids makes him able to get that information. He threatens to hurt me through the children” and “My partner has been stalking and harassing me. I have restraining orders and a protective order; both have been breached.”

Three women feared their ex-partners could kill them, noting previous serious assaults or threats of homicide, for example, “Is he going to show up drunk? Will he hurt my daughter? I know what he's capable of. If push comes to shove, he would take my life”; “He tried to kill me once before. My baby was with me, I was all bloody”; and “He has threatened to kill me. I believe he would. Stalking me for the last six years and, now that he knows where I live, it is worse.”

Sixteen comments (14.8%) were specific to child safety, for example, “I worry that he is neglectful. He does not take care of his children properly. I’m worried about emotional abuse”; “When they are with him, in case he's not taking his meds for depression or being suicidal or drinking”; “He follows, terrorizes, and threatens my children even though he lives 50 km away. He drives around the house when we are home alone”; and “Safety when alone with dad. Four-year-old come home with bruises, cuts, and scrapes. He is witnessing violence, and I can’t protect him (she reported to Child Protective Services).” Four mothers were concerned that their ex-partner might abduct his children, for example, “He told people and me that he will run with the children.” Altogether, the mothers’ comments reflect major concerns about safety for themselves and their children.

*The impact of father's regular contact on mothers.* Regarding comments about the impact of the regular visits on the mothers, over half (57 of 105, 54.3%) found them difficult or worrisome, for example, “Very difficult to have kids go somewhere that they’re being emotionally abused. I can tell by the way the kids act out when they come back” and “The visits just about killed me in the beginning. He never cared about kids until we separated; being with them is just about saving some money.” Another 21 (27.6%) had no concerns about the visits, for example, “In a lot of ways, it's good because I know he can’t take off with them” and “It doesn’t bother me. I would love to see him come five days a week, but his work always comes first. I am glad that he makes an effort.” Smaller proportions commented on the irregularity of the visits being problematic (11, 10.5%), difficulties associated with being a single parent (9, 8.6%), and no impact (7, 6.7%). For mothers, then, a sizable proportion experienced a positive impact, while unfortunately, the impact was negative for the majority.

*The impact of father's regular contact on children.* More than two fifths (49, 41.9%) of the mother's 117 comments described their children as sad or upset, for example, “The children get frustrated because of different rules. I notice behaviour changes after the visits” and “I asked my daughter if she missed her father and she covered my mouth with her hand, making me think she doesn’t want to talk about it. She's only three.” Some noted a significant toll on their children's mental health, for example, “My son internalizes everything, he's been told by the father not to talk about feelings. He's got low self-esteem, problems in school, has bowel and urinary accidents, nervous ticks. We’ve had to get counseling for him talking about suicide after these visits.” A smaller number (17, 14.5%) described their children as having angry, acting-out behaviors, for example, “He has torn up the children. He uses them as weapons. Older son abuses me now, displaying anger. Kids are in counseling”; “Causes him to have behavioral problems—aggressive, doesn’t listen. He has temper tantrums, sleep problems, restlessness, nightmares”; and “It's about a year since visits started—son crying in class, chews nails, can’t concentrate. Acting out in angry ways.” Five mothers mentioned that their children were in counseling, however, an ex-partner of another refused to give consent.

In contrast, almost one-quarter of the mother's comments (28, 23.9%) described their children as amenable to the visits, for example, “They look forward to it”; “At first, they weren’t so happy, my youngest son was depressed. He was used to the whole family being together, but now he's OK”; and “My son is happy again. Chattering more—sings to himself. He is happy to see Dad now—not so scared of him.” A small number (10, 8.5%) reported no impact, three because the children were too young. Smaller proportions of the children were described as missing their fathers (6, 5.1%), reacting to irregular visits (4, 3.4%), and wanting no further contact with fathers (3, 2.6%).

### Summary Across Types of Father–Child Contact

Collapsing the “no impact” and “safe/happy” categories for the mothers, more than half of the mothers with no contact (54.8%) and slightly more than two fifths of those with sporadic contact (44.7%) reported positive or neutral effects of the contact arrangements. This compares to only a little more than one-third of the mothers (34.3%) whose ex-partners visited their children regularly. That over half of the mothers (54.3%) were worried or had difficulties with regular father–child visits and that 41.9% perceived their children to be sad or upset and another 14.5% reported children who were angry or acting out supports the conclusion that ongoing contact with a father who has abused their mothers remains concerning for a large proportion of the mothers and children. A small number of cases were of significant concern, either because of IPV or child abuse risk or the extreme reactions of the children and significant toll on their mental and physical health. Even with no contact with ex-partners, seven mothers (6.7% of 105) commented about significant concerns about their children, and seven mothers whose children had sporadic contact and 23 with regular father–child visits (30 of 176, 17.1%) reported concerning behaviors on the part of the ex-partners toward them, their children, or both.

*Supervised visits.* An issue that emerged from the mother's comments was with respect to supervised father/child visits. Among the women whose partners had no contact with their children, three mentioned that supervised visits had been a possibility but were not enacted, for example, “He is court ordered to have no contact with her and the kids. However, he can access the children through supervised visitation through a third party of my choosing. He hasn’t seen the kids in about three years.” Two others mentioned that their ex-partners had participated but then stopped attending scheduled supervised visits.

Of fathers who visited only sporadically, eight mothers mentioned supervised visits (four as a possibility in future), for example, “He was to have supervised visits only (I insisted on that). But he hasn’t arranged for a supervisor like he was supposed to, so visits haven’t occurred” and “She had it made court-ordered that if he did pick up the kids; it would be at her mom's, a supervised exchange.” In several other cases, the fathers did not use the supervised visit option, for example, “Their dad is allowed to visit with supervision (I cannot supervise). He does not visit at all” and “Interim custody with supervised visits only but ex hasn’t utilized that.” Only two fathers seemed to have used the supervision option: “Father visits, supervised (as dictated by me not the court). He visits approximately once every three months” and “He is only allowed to see my daughter in supervised day visits because of abuse and drug use.”

Eighteen mothers whose ex-partners had regular contact with their children mentioned supervised visits. For nine, the supervised visits seemed routine, for example, “He hasn’t been doing any long-term care, but he has a supervised visit once per week”; “He can only meet with his daughter in [social service agency]”; and “He has supervised visits two days a week for two hours; anytime with 24 h’ notice. Supervisor is another family member, or friend and me.”

Five mothers reported that the fathers started with supervised visits that changed to unsupervised: “He initially only had supervised visits because of abuse—now he has them unsupervised”; “Supposed to be supervised visitation with his mom—now he comes here to visit son and baby”; and “He had two supervised visits. Now he gets the children every other weekend Friday 6 pm until Sunday 4 pm.” Another changed from no contact to supervised visits. For three it was recommended, “My children/s counselor recommends safe visitation program/supervised visits,” recommended but not enacted, “Brain injury specialist, recommended supervised visit.” A final mother had hoped for supervised visits, but these were not arranged.

## Discussion

It was important to not only examine the mother's concerns about the impact of father's visits on themselves or their children, but also to view these in the context of the significant IPV that a number had experienced, with threats not only to their own safety but concerns about child abductions and child abuse during visitation. Without such context, the effects could be considered as merely similar to mother and child responses to visitation in families with no history of IPV (Elam et al., 2016).

The serious nature of the IPV and ongoing inappropriate and sometimes threatening behaviors from some fathers explains the concerning emotional reactions to father contact/no contact from many women and their children. Moreover, mothers’ fears for their children's safety during visits with their ex-partners are indeed appropriate—children have been murdered during access visits with their fathers (David et al., 2017). In fact, in our parallel Healing Journey analysis on IPV and custody arrangements, one mother's Indigenous son died from a physical assault by his father, who was awarded full custody (Tutty et al., under review).

In the context of IPV, no contact between a father and his children was sometimes related to his history of physical abuse. Thirty-seven percent of the mothers reported no child contact, slightly more than the 30% in Hunter and Graham-Bermann's (2013) study with a similar sample size (219 compared to 280) and 32% in Forssell and Cater's (2015) study of 165 children. It is perhaps no surprise that the fathers with no contact were reported to have used significantly more serious physical abuse against the mothers when they were together. Moreover, 26 of the 104 men were either in jail or had no contact or restraining orders against them, both being further indications of the possible dangers of allowing them to spend time with their children.

Mothers also identified problems when contact between fathers and children occurred. The father's irregularity or inconsistency with respect to child visits was a problem for mothers and children in a number of cases, similar to previous research (Holt, 2015, 2017; Reece, 2006). A relatively small proportion of the mother's comments (37 of 280, 13.2%) described concerning, ongoing IPV, child abuse, or threats of this, however, these cases merit close attention due to the potential harm. The mothers seemed to have few options to address these situations, as police intervention or residing in a VAW shelter were not appropriate at the time. Furthermore, restraining or protection orders were viewed skeptically by a number of mothers, similar to other research on women whose partners abuse them (Messing et al., 2021).

None of the mothers reported the father's using specialized supervised visitation centers (Birnbaum & Allagia, 2006; Harrison, 2008; Morrison & Wasoff, 2012; Sears et al., 2022), despite the fact that some lived in communities with such programs (Tutty et al., 2006). Rather, most of their supervised visits were conducted with friends or family members supervising the exchanges or the visits. Even so, a number struggled to continue the supervised visits, with little support from community agencies. The viability of supervised visits after IPV needs further study.

### Implications for Practitioners

Most interventions for IPV focus on mothers in crisis and in the process of separating from abusive partners. Clearly, the “crisis” for many mothers and their children extends far beyond the point of marital separation. An analysis of the larger sample of 660 women from the Healing Journey study (Tutty, 2023) noted that the majority had received counseling at some point, with counselors in VAW shelters being rated as the most helpful. Much of this support was in the early days of separating from partners and not when mothers were dealing with ongoing concerns about father/child contact that may extend for years. In the current study, even though a small number of mothers described their children as exhibiting concerning symptoms of anger or depression, only seven (2.5%) mentioned that their children had received counseling, perhaps because of the limited availability of such services.

Mothers and children with negative ongoing psychological reactions and realistic concerns about fathers’ contact visits might be supported in interventions for mothers (i.e., Austin et al., 2019; Howell et al., 2015) and children exposed to IPV (i.e., Graham-Bermann & Hughes, 2003). Jouriles et al. (2018) evaluated a program entitled “Project Support,” a post-VAW shelter program offering parenting support to abused women who did not intend to return to abusive ex-partners. The evaluation concluded that reduced father–child contact was associated with fewer conduct behavior problems for girls but not for boys.

Specialized initiatives for children such as the “Speaking for Ourselves” program at the Calgary YWCA (Fotheringham et al., 2013) provide both a trauma therapist and a lawyer for children whose parents are in high conflict custody and access litigation. This evaluation reported that before entering the program, children were highly traumatized on average, and program participation reduced some symptoms to below clinical levels. Implementing similar initiatives for children who seldom have a voice in custody battles is recommended. Despite these exemplary interventions, few non-VAW shelter programs or IPV-specific counseling services are available that address problems associated with father/child contact. More proactive interventions by practitioners to both alert women to their children's possibly negative reactions to contact with their fathers and to offer counseling specific to continued interactions with ex-partners are needed.

Although beyond the scope of this analysis, there are important legal concerns worth noting. Family courts tend to make decisions based on the presumption that children benefit from maximum contact with both parents; therefore, are likely to award at least some kind of contact to fathers—including ones who have a history of IPV (Macdonald, 2016, 2017; Neilson, 2014). Recognizing the harm that children may suffer under dangerous circumstances, scholars and legal advocates have expressed serious concern and urged judicial actors to engage in thoughtful analysis that properly prioritizes children, such as adopting a child right's analysis when making decisions, including court-ordered visitation.

Many of the mothers in our study commented that their children suffered significant emotional and behavioral impacts from having unwanted contact with their fathers. Surely, allowing children to have a voice in these important decisions would help buffer them from these deleterious effects. This could serve as a protective factor and increase their feelings of personal control and enhanced resiliency (Boshier & Steel-Baker, 2007; Fotheringham et al., 2013). Family courts are urged to consider the potential harm and danger of contact between children and abusive fathers, including listening to children's wishes about such contact.

### Study Limitations and Strengths

Unlike qualitative studies on child contact after IPV that used in-depth interviews with mothers (Coy et al., 2015; Elizabeth, 2017; Hoffart et al., 2022; Holt, 2015; Hunter & Graham-Bermann, 2013; Morrison, 2015; Thiara & Humphreys, 2017; Tolmie et al., 2009), the mother's comments in this study were in response to survey questions about custody arrangements and visitation, which were only one of a long series of questions about issues faced by women abused by intimate partners. This limitation is somewhat mitigated by the survey questions being open-ended, so that the women were free to determine the focus of their answers rather than addressing a list of possible issues. As well, the comments provided clear snapshots and in-depth descriptions of the women's concerns about father–child contact.

As with most secondary analyses, the data were collected some years before. Processes and laws about child custody and visitation may have changed since data collection in 2004, although we could find no major revisions to this in the three Canadian provinces since then (Hoffart et al., 2022).

Regarding study strengths, the current analysis provides information about visitation on a large sample of women whose ex-partners had abused them when they lived together (*N* = 280) and included a group of mothers whose children had no father contact and some with only sporadic contact. Few studies on the nature of father–child visitation after IPV are quantitative as is the current study (exceptions include Holt, 2011; Jouriles et al., 2018; Morrill et al., 2005). Furthermore, a paucity of studies examines circumstances when fathers have no contact with children (exceptions include Forssell & Cater, 2015; Hunter & Graham-Bermann, 2013; Jouriles et al., 2018; Lapierre et al., 2022), and none differentiates regular from sporadic contact, although we found this to be consequential.

Finally, the largest group of women was Indigenous, an important population in the Canadian context, and one about which custody and visitation arrangements are lacking. That the nature of the father–child visits (contact vs. no contact) did not differ from the White or Visible Minority mothers adds to our understanding of the circumstances of Indigenous women.

## Conclusion

That children and youth are often negatively impacted by separation and divorce is not debated (Elam et al., 2016). When the context of the parent's previous relationship includes significant IPV, ongoing contact and exchanges between fathers and children may include continued threats of IPV, child abuse, and even child homicide. Resources specific to these circumstances are lacking and typical responses to IPV such as restraining or protection orders, police intervention, or VAW shelters are seldom appropriate. Mothers re-litigating custody as a strategy to resolve visitation problems is unlikely, as most have limited financial resources and their previous custody battles were often emotionally fraught (Tutty et al., under review).

Interventions focused on the post-separation/divorce period for both mothers and children are clearly needed. Among these, family courts should consider prioritizing children's voices in determining appropriate contact with fathers and recognizing the problems and dangers associated with contact with fathers in the context of a history of IPV.

## References

[bibr1-10778012231225232] Ateah C. Radtke H. L. Tutty L. M. Nixon K. Ursel E. J. (2019). Mothering, guiding and responding to children: A comparison of women abused and not abused by intimate partners. Journal of Interpersonal Violence, 34(15), 3107–3126. 10.1177/0886260516665109 27550444

[bibr2-10778012231225232] Austin A. E. Shanahan M. E. Barrios Y. V. Macy R. J. (2019). A systematic review of interventions for women parenting in the context of intimate partner violence. Trauma, Violence, & Abuse, 20(4), 498–519. 10.1177/1524838017719233 29333985

[bibr3-10778012231225232] Birnbaum R. Allagia R. (2006). Supervised visitation: A call for a second generation of research. Family Court Review, 44(1), 119–134. 10.1111/j.1744-1617.2006.00071.x

[bibr4-10778012231225232] Boshier P. Steel-Baker D. (2007). Invisible parties: Listening to children. Family Court Review, 45(4), 548–559. 10.1111/j.1744-1617.2007.00170.x

[bibr5-10778012231225232] Brownridge D. A. Chan K. L. Hiebert-Murphy D. Ristock J. Tiwari A. Leung W. C. Santos S. C. (2008). The elevated risk for non-lethal post-separation violence in Canada: A comparison of separated, divorced, and married women. Journal of Interpersonal Violence, 23(1), 117–135. 10.1177/0886260507307914 18087035

[bibr6-10778012231225232] Cohen J. (1988). Statistical power analysis for the behavioral sciences (2nd ed.). Lawrence Erlbaum Associates.

[bibr7-10778012231225232] Coy M. Scott E. Tweedale R. Perks K. (2015). “It’s like going through the abuse again”: Domestic violence and women and children’s (un)safety in private law contact proceedings. Journal of Social Welfare & Family Law, 37(1), 53–69. 10.1080/09649069.2015.1004863

[bibr8-10778012231225232] David R. Olszowy L. Reif K. Saxton M. Campbell M. Dubé M. Dawson M. Jaffe P. (2017). *Children and domestic homicide: Understanding the risks* (Domestic Homicide Brief (3)). Canadian Domestic Homicide Prevention Initiative. http://cdhpi.ca/sites/cdhpi.ca/files/Brief_3-Final.pdf

[bibr9-10778012231225232] Dawson M. Bunge V. P. Balde T. (2009). National trends in intimate partner homicides: Explaining declines in Canada, 1976 to 2001. Violence Against Women, 15(3), 276–306. 10.1177/1077801208330433 19158316

[bibr10-10778012231225232] DeKeseredy W. S. Rogness M. Schwartz M. D. (2004). Separation/divorce sexual assault: The current state of social scientific knowledge. Aggression and Violent Behavior, 9(6), 675–691. 10.1016/j.avb.2003.08.004

[bibr11-10778012231225232] DeRiviere L. (2014). The healing journey: Intimate partner abuse and its implications in the labour market. Fernwood Press and RESOLVE.

[bibr12-10778012231225232] Elam K. K. Sandler I. Wolchik S. Tein J. (2016). Non-residential father–child involvement, interparental conflict and mental health of children following divorce: A person-focused approach. Journal of Youth and Adolescence, 45(3), 581–593. 10.1007/s10964-015-0399-5 26692236 PMC4749464

[bibr13-10778012231225232] Elizabeth V. (2017). Custody stalking: A mechanism of coercively controlling mothers following separation. Feminist Legal Studies, 25(1), 185–201. 10.1007/s10691-017-9349-9

[bibr14-10778012231225232] Elizabeth V. Gavey N. Tolmie J. (2012). The gendered dynamics of power in disputes over the post-separation care of children. Violence Against Women, 18(4), 459–481. 10.1177/1077801212452049 22761171

[bibr15-10778012231225232] Elo S. Kyngäs H. (2008). The qualitative content analysis process. Journal of Advanced Nursing, 62(1), 107–115. 10.1111/j.1365-2648.2007.04569.x 18352969

[bibr16-10778012231225232] Field A. (2009). Discovering statistics using SPSS (3rd ed.). Sage Publications.

[bibr17-10778012231225232] Fleury-Steiner R. E. Miller S. L. Maloney S. Postel E. B. (2016). “No contact, except…”: Visitation decisions in protection orders for intimate partner abuse. Feminist Criminology, 11(1), 3–22. 10.1177/1557085114554259

[bibr18-10778012231225232] Forssell A. M. Cater A. (2015). Patterns in child-father contact after parental separation in a sample of child witnesses to intimate partner violence. Journal of Family Violence, 30(3), 339–348. 10.1007/s10896-015-9673-2

[bibr19-10778012231225232] Fotheringham S. F. Dunbar J. Henskey D. (2013). Speaking for themselves: Hope for children caught in high conflict custody and access disputes involving domestic violence. Journal of Family Violence, 28(4), 311–324. 10.1007/s10896-013-9511-3

[bibr20-10778012231225232] Graham-Bermann S. A. Hughes H. M. (2003). Intervention for children exposed to interparental violence (IPV): Assessment of needs and research priorities. Clinical Child and Family Psychology Review, 6(3), 189–204. 10.1023/A:1024962400234 14620579

[bibr21-10778012231225232] Graneheim U. H. Lundman B. (2004). Qualitative content analysis in nursing research: Concepts, procedures and measures to achieve trustworthiness. Nurse Education Today, 24(2), 105–112. 10.1016/j.nedt.2003.10.001 14769454

[bibr22-10778012231225232] Hardesty J. L. Ganong L. H. (2006). How women make custody decisions and manage co-parenting with abusive former husbands. Journal of Social and Personal Relationships, 23(4), 543–563. 10.1177/0265407506065983

[bibr23-10778012231225232] Harrison C. (2008). Implacably hostile or appropriately protective? Women managing child contact in the context of domestic violence. Violence Against Women, 14(4), 381–405. 10.1177/1077801208314833 18359876

[bibr24-10778012231225232] Hayes B. (2012). Abusive men’s indirect control of their partner during the process of separation. Journal of Family Violence, 27(4), 333–344. 10.1007/s10896-012-9428-2

[bibr25-10778012231225232] Hayes B. E. (2017). Indirect abuse involving children during the separation process. Journal of Interpersonal Violence, 32(19), 2975–2997. 10.1177/0886260515596533 26216243

[bibr26-10778012231225232] Heaton J. (2008). Secondary analysis of qualitative data: An overview. Historical Social Research/Historische Sozialforschung, 33(3), 33–45. https://www.jstor.org/stable/20762299

[bibr27-10778012231225232] Hegarty K. Bush R. Sheehan M. (2005). The composite abuse scale: Further development and assessment of reliability and validity of a multidimensional partner abuse measure in clinical settings. Violence and Victims, 20(5), 529–547. 10.1891/088667005780927548 16248489

[bibr28-10778012231225232] Hoffart R. Dyer K. Bresch L. Haller A. Warren S. Nixon K. (2022). Caught in the middle: Children’s involvement in the court process as it relates to intimate partner violence. RESOLVE Manitoba. https://www.umanitoba.ca/sites/resolve/files/2023-03/Caught%20in%20the%20Middle%20Final%20Report_2022%20_0.pdf

[bibr29-10778012231225232] Holt S. (2011). Domestic abuse and child contact: Positioning children in the decision-making process. Child Care in Practice, 17(4), 327–346. 10.1080/13575279.2011.596817

[bibr30-10778012231225232] Holt S. (2015). Post-separation fathering and domestic abuse: Challenges and contradictions. Child Abuse Review, 24(3), 210–222. 10.1002/car.2264

[bibr31-10778012231225232] Holt S. (2017). Domestic violence and the paradox of post-separation mothering. British Journal of Social Work, 47(7), 2049–2067. 10.1093/bjsw/bcw162

[bibr32-10778012231225232] Howell K. H. Miller L. E. Lilly M. M. Burlaka V. Grogan-Kaylor V. Graham-Bermann S. A. (2015). Strengthening positive parenting through intervention: Evaluating the Moms’ Empowerment Program for women experiencing intimate partner violence. Journal of Interpersonal Violence, 30(2), 232–252. 10.1177/0886260514533155 24832954

[bibr33-10778012231225232] Humphreys C. Harrison C. (2003). Focusing on safety: Domestic violence and the role of child contact centres. Child and Family Law Quarterly, 15(3), 237–253. https://ssrn.com/abstract=2030938

[bibr34-10778012231225232] Hunter E. C. Graham-Bermann S. A. (2013). Intimate partner violence and child adjustment: Moderation by father contact? Journal of Family Violence, 28(5), 435–444. 10.1007/s10896-013-9517-x

[bibr35-10778012231225232] Jouriles E. N. Rosenfield D. McDonald R. Vu N. L. Rancher C. Mueller V. (2018). Children exposed to intimate partner violence: Conduct problems, interventions, and partner contact with the child. Journal of Clinical Child & Adolescent Psychology, 47(2), 397–409. 10.1080/15374416.2016.1163706 27359091 PMC6815933

[bibr36-10778012231225232] Katz E. Nikupeteri A. Laitinen M. (2020). When coercive control continues to harm children: Post-separation fathering, stalking and domestic violence. Child Abuse Review, 29(4), 310–324. 10.1002/car.2611

[bibr37-10778012231225232] Lapierre S. Côté I. Lessard G. (2022). “He was the king of the house:” children’s perspectives on the men who abused their mothers. Journal of Child Custody, 19(3–4), 244–260. 10.1080/26904586.2022.2036284

[bibr38-10778012231225232] Long-Sutehall T. Sque M. Addington-Hall J. (2010). Secondary analysis of qualitative data: A valuable method for exploring sensitive issues with an elusive population? Journal of Research in Nursing, 16(4), 335–344. 10.1177/1744987110381553

[bibr39-10778012231225232] Macdonald G. (2017). Hearing children’s voices? Including children’s perspectives on their experiences of domestic violence in welfare reports prepared for the English courts in private family law proceedings. Child Abuse & Neglect, 65(1), 1–13. 10.1016/j.chiabu.2016.12.013 28092738

[bibr40-10778012231225232] Macdonald G. S. (2016). Domestic violence and private family court proceedings promoting child welfare or promoting contact? Violence Against Women, 22(7), 832–852. 10.1177/1077801215612600 26567294

[bibr41-10778012231225232] Messing J. T. Bagwell-Gray M. E. Ward-Lasher A. Durfee A. (2021). ‘Not bullet proof’: The complex choice not to seek a civil protection order for intimate partner violence. International Review of Victimology, 27(2), 173–195. 10.1177/0269758021993338

[bibr42-10778012231225232] Morrill A. C. Dai J. Dunn S. Sung I. Smith K. (2005). Child custody and visitation decisions when the father has perpetrated violence against the mother. Violence Against Women, 11(8), 1076–1107. 10.1177/1077801205278046 16043586

[bibr43-10778012231225232] Morrison F. (2015). ‘All over now?’ the ongoing relational consequences of domestic abuse through children’s contact arrangements. Child Abuse Review, 24(4), 274–284. 10.1002/car.2409

[bibr44-10778012231225232] Morrison F. Wasoff F. (2012). Child contact centers and domestic abuse victim safety and the challenge to neutrality. Violence Against Women, 18(6), 711–720. 10.1177/1077801212454257 22855127

[bibr45-10778012231225232] Nassaji H. (2015). Qualitative and descriptive research: Data type versus data analysis. Language Teaching Research, 19(2), 129–132. 10.1177/1362168815572747

[bibr46-10778012231225232] Neergaard M. A. Olesen F. Andersen R. S. Sondergaard J. (2009). Qualitative description: The poor cousin of health research? BMC Medical Research Methodology, 9(1), 52–56. 10.1186/1471-2288-9-52 19607668 PMC2717117

[bibr47-10778012231225232] Neilson L. C. (2014). Enhancing safety: When domestic violence cases are in multiple legal systems (criminal, family, child protection): A family law, domestic violence perspective. Department of Justice. https://www.justice.gc.ca/eng/rp-pr/fl-lf/famil/enhan-renfo/neilson_web.pdf

[bibr48-10778012231225232] Nixon K. L. Tutty L. M. Radtke H. L. Ateah C. Ursel E. J. (2017). Protective strategies of mothers abused by intimate partners: Rethinking the deficit model. Violence Against Women, 23(11), 1271–1292. 10.1177/1077801216658978 27535939

[bibr49-10778012231225232] Rea L. M. Parker R. A. (1992). Designing and conducting survey research. Jossey-Bass.

[bibr50-10778012231225232] Reece H. (2006). UK women’s groups’ child contact campaign: ‘so long as it is safe’. Child and Family Law Quarterly, 18(4), 538–561. https://ssrn.com/abstract=1967567

[bibr51-10778012231225232] Rezey M. L. (2020). Separated women’s risk for intimate partner violence: A multiyear analysis using the national crime victimization survey. Journal of Interpersonal Violence, 35(5–6), 1055–1080. 10.1177/0886260517692334 29294656

[bibr52-10778012231225232] Rosen L. N. O’Sullivan C. S. (2005). Outcomes of custody and visitation petitions when fathers are restrained by protection orders. Violence Against Women, 11(8), 1054–1075. 10.1177/1077801205278045 16043585

[bibr53-10778012231225232] Sandelowski M. (2000). Combining qualitative and quantitative sampling, data collection, and analysis techniques in mixed-method studies. Research in Nursing & Health, 23(3), 246–255. 10.1002/1098-240X(200006)23:3<246::AID-NUR9>3.0.CO;2-H 10871540

[bibr54-10778012231225232] Sears J. S. Wilfong J. Diemer M. (2022). Supervised visitation: State of the research. Families in Society: The Journal of Contemporary Social Services, 194(4). 10.1177/1044389422111

[bibr55-10778012231225232] Stark E. (2007). Coercive control: How men entrap women in personal life. Oxford University Press.

[bibr56-10778012231225232] Sullivan-Bolyai S. Bova C. Harper D. (2005). Developing and refining interventions in persons with health disparities: The use of qualitative description. Nursing Outlook, 53(3), 127–133. 10.1016/j.outlook.2005.03.005 15988449

[bibr57-10778012231225232] Thiara R. Humphreys C. (2017). Absent presence: The ongoing impact of men’s violence on the mother-child relationship. Child and Family Social Work, 22(1), 137–145. 10.1111/cfs.12210

[bibr58-10778012231225232] Tolmie J. Elizabeth V. Gavey N. (2009). Raising questions about the importance of father contact within current family law practices. New Zealand Law Review, 4(4), 659–694.

[bibr59-10778012231225232] Tutty L. M. (2023). “Looking back, the programs kept me alive”: Women’s impressions of counselling for intimate partner violence. Women & Therapy. 10.1080/02703149.2023.2167309

[bibr60-10778012231225232] Tutty L. M. Barry L. Weaver-Dunlop J. Barlow A. Roy M. (2006). Supervised visitation and exchange centres for domestic violence: An environmental scan. RESOLVE Alberta. https://nursing.ucalgary.ca/sites/default/files/teams/13/Supervised%20visitation%20and%20exchange%20centres%20for%20domestic%20violence%20An%20environmental%20scan.pdf

[bibr61-10778012231225232] Tutty L. M. Radtke H. L. Ateah C. Ursel E. J. Thurston W. E. Hampton M. Nixon K. L. (2021a). The complexities of intimate partner violence: Mental health, physical disabilities and child abuse history for white, indigenous, and other visible minority Canadian women. Journal of Interpersonal Violence, 36(3–4), 1208–1232. 10.1177/0886260517741210 29294979

[bibr62-10778012231225232] Tutty L. M. Radtke H. L. Nixon K. L. (2023). “He tells people that I am going to kill my children”: Post-separation coercive control in men who perpetrate IPV. Violence Against Women. 10.1177/10778012231166408 PMC11316341 37006166

[bibr63-10778012231225232] Tutty L. M. Radtke H. L. Nixon K. L. (under review). “It just rips my heart out”: Child custody dispositions after women leave abusive partners. 10.1177/10778012251329259 PMC13018247 40130523

[bibr64-10778012231225232] Tutty L. M. Radtke H. L. Thurston W. E. Ursel E. J. Nixon K. L. Hampton M. Ateah C. (2021b). A longitudinal study of the well-being of Canadian women abused by intimate partners: A healing journey. Journal of Aggression, Maltreatment and Trauma, 30(9), 1125–1147. 10.1080/10926771.2020.1821852

[bibr65-10778012231225232] Varcoe C. Irwin L. G. (2004). “If I killed you, I’d get the kids”: Women’s survival and protection work with child custody and access in the context of woman abuse. Qualitative Sociology, 27(1), 77–99. 10.1023/B:QUAS.0000015545.82803.90

[bibr66-10778012231225232] World Health Organization. (2021). *Violence Against Women prevalence estimates, 2018: Global, regional and national prevalence estimates for intimate partner violence against women and global and regional prevalence estimates for non-partner sexual violence against women* . https://www.who.int/publications/i/item/9789240022256

